# Five Questions about Viral Trafficking in Neurons

**DOI:** 10.1371/journal.ppat.1002472

**Published:** 2012-02-16

**Authors:** L. W. Enquist

**Affiliations:** Department of Molecular Biology, Princeton University, Princeton, New Jersey, United States of America; Columbia University, United States of America

## Why Are We Interested in This Subject?

One of the most exciting areas in biology is the nervous system and how it works. Viral infections of the nervous system have provided exceptional insight at many levels, from pathogenesis to basic biology. The nervous system has evolved rather complicated barriers that facilitate access to nutrients and contact with the outside world, but block entry of pathogens and toxins [1]. However, when these barriers are reduced for any number of reasons, nervous system infections are possible. When they occur, they can be devastating and, even with good antiviral drugs, difficult to manage. Viral infections can enter the brain via the blood (e.g., HIV, various encephalitis viruses) or by spread inside neurons from the body surface (e.g., rabies and alpha herpes viruses) [2], [3]. In vertebrates, the nervous system comprises a peripheral collection of neurons (the peripheral nervous system, PNS) and a central set found in the brain and spinal cord (the central nervous system, CNS). While neurons are central players in neurobiology, it is important to realize that the majority of cells that comprise the nervous system are highly specialized, non-neuronal cells (e.g., different types of glial cells) [4]. Cells of the immune system also engage with and signal to the PNS to affect changes in the CNS [5].

We will focus on neurons, despite the other cellular complexity, because neurons provide direct avenues for viral infection. Recognition that viral infection follows nerve pathways enabled the development of viruses for neuronal circuit tracing [6]–[8].

## What Common Virus Families Infect the Nervous System?

The initial interaction of host and virus particles in peripheral tissue prior to nervous system infection can be complex, often involving significant productive replication and spread among a variety of cell types, including those present at mucosal surfaces as well as those of the immune and nervous systems. On the other hand, some viruses (e.g., rabies virus) are neurotropic and invade neurons at neuromuscular junctions with limited peripheral replication in other cell types. Despite complicated initial interactions, members of many virus families can infect the CNS from the blood or from the PNS, including Picornaviridae, Rhabdoviridae, Paramyxoviridae, Togaviridae, Retroviridae, Flaviviridae, Arenaviridae, and Polyomaviridae [3]. Interestingly, members of the alphaherpesvirinae subfamily of the Herpesviridae establish quiescent, reactivatable infections of PNS neurons of their natural hosts and rarely cause CNS infections [9].

## Why Are Neurons Used as Pathways for Disseminating Infection?

**Figure 1:**
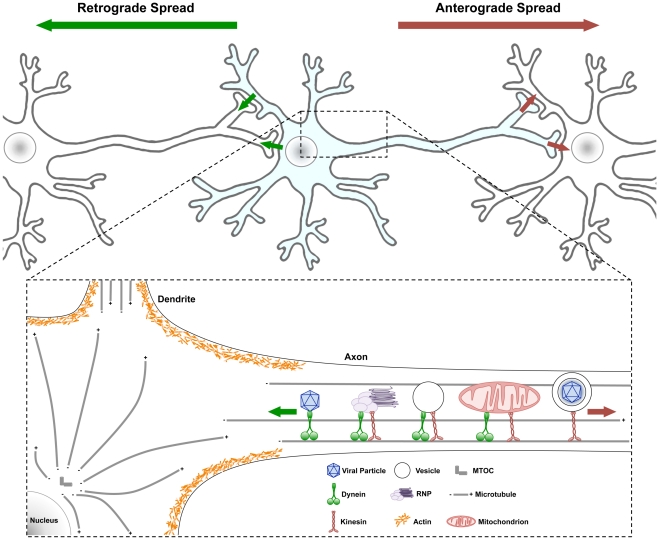
Traffic patterns in axons of viral infected neurons. Neurons have a cell body containing core cytoplasmic components and a nucleus with all the transcription machinery. Neurons also have highly specialized extensions of their cell body cytoplasm called dendrites and axons. Usually neuronal cell bodies extend a single axon over long distances to connect with other neurons and cells. Action potentials initiate with voltage gated ion channels located in the proximal segment where the axons extend from the cell body. As ion channels open and close, action potentials move down axonal membranes to sites of synaptic contact where neurotransmitters are released and signal to postsynaptic cells. (Top) Infection spreading from neuron to neuron can be in the anterograde direction (red arrow, from infected pre-synaptic neuron to connected post-synaptic neuron) or in the retrograde direction (green arrow, from the infected post-synaptic neuron to the connected presynaptic neuron). (Bottom) Some structures and organelles that move in axons on microtubules using dynein (from + to − end) or kinesin (from − to + end) motors are illustrated. Some complexes have both motors, which can be differentially controlled. Obviously, these structures and organelles also are transported in dendrites and within the cell body on microtubules as well. Actin cytoskeletal structures are shown only schematically around the cell body cortex, and the various myosin motors are not illustrated [22].

PNS neurons provide the interface with the outside world by sending and receiving information to and from the CNS. PNS neurons often are only one synapse away from the CNS. Others, including neurons in cranial nerves, may have cell bodies in the CNS and axon terminals near the body surface. Neurons are highly specialized cells that expend a great deal of energy maintaining their elaborate structures through the trafficking of all their cellular constituents. The neuronal cell body contains the core cellular components and the nucleus with all the cellular RNA transcription machinery. These cells also have highly specialized extensions of their cell body cytoplasm called dendrites and axons (Figure 1). Dendrites can be branched (sometimes extensively), and make connections (synapses) with axons from other neurons. Axons are single extensions from the neuronal cell body that cover long distances to connect with other neurons and cells. Axons can branch and can be so long that they contain the majority of neuronal cytoplasm [10]. They are the wires of the nervous system literally conducting electrical currents to and from the extremes of the body. These same conduits are utilized for the long distance spread of viral infection within the host organism.

## Why Is Traffic in Axons Important to Understand?

Most neurons are terminally differentiated and never divide. Nevertheless, these cells are not quiescent: their metabolism is phenomenally active, they are relatively resistant to many insults, and their cytoplasm is in constant motion. The coordinated movement of components inside neurons is termed “traffic” and it occurs on the neuronal cytoskeletal architecture comprised of actin, microtubules, and other intermediate filaments driven by motors such as the myosins, dyneins, and kinesins (Figure 1). Traffic of anything implies motion, and motion without regulation leads to cacophony not a symphony. The homeostasis and responsiveness of a neuron involves moving cellular components and organelles to and from the extremes of a neuron (e.g., dendrites and axons). Such movement must be coordinated with events that can be millimeters to meters away from the cell body. Even messenger RNAs, as part of ribonucleoprotein complexes, are moved long distances to specialized sites in dendrites and axons for relatively instantaneous synthesis of new proteins required far away from the cell body [11]. Maintaining the health and function of the cytoplasm in axons is an exercise in sensing local environments, and long distance communication coupled with transport of cellular organelles [12], [13]. Defects in axonal transport are implicated in an increasing number of neuropathogenic disorders, including Parkinson's Disease, Alzheimer's Disease, Charcot Marie Tooth Disease, Huntington's Disease, and Lou Gehrig's Disease (ALS) [10]. How cytoplasmic traffic is coordinated in uninfected and infected neurons presents challenging problems.

## How Does Viral Traffic Move in Neurons?

We know of no viral genome that encodes analogs of cellular motors or cytoskeletal structures. Viral proteins and complex structures such as nucleocapsids or enveloped particles must engage the neuronal transport machinery to move about the cell [9], [14], [15]. Neuroinvasive viruses must express viral proteins that repurpose, redirect, or embellish existing neuronal traffic control to replicate and disseminate viral material to perpetuate the infection [15]–[18]. Moreover, viral proteins move in the context of extensive endogenous neuronal traffic whose cargo must continue to be sorted to cell body, dendritic, and axonal compartments to keep the neuron alive [19]. In general, neuronal and viral proteins that direct traffic must distinguish among different classes of cargo (Figure 1). Viral proteins engage motors directly, modify regulatory pathways to alter transport machinery, and in the process, probably alter organelle traffic [2], [16].

The majority of long distance, intracellular movement of cargo, including that of endocytic/exocytic vesicles, organelles, and chromosomes, occurs on microtubules [15]. Microtubules form relatively stiff polymers comprised of heterodimers of α and β-tubulin. Microtubules have a dynamic “plus” end that can grow and shrink rapidly while the “minus” end is tethered to the microtubule-organizing center and is relatively stable. In axons, microtubules are strictly oriented with the minus end toward the cell body and the plus end toward axon terminals (or growth cones). Interestingly, many or all of the microtubules found in axons are no longer connected to the microtubule organizer region (MTOC). They are nucleated by the MTOC in the cell body and then transported into and along axons by slow axonal transport. The microtubules in axons are not single ultra long polymers, but are discontinuous, overlapping bundles. Maintaining the integrity of the microtubule highway in axons is paramount for neuronal homeostasis and, not surprisingly, for optimal viral traffic.

Two classes of molecular motors govern directional transport along microtubules. Plus-end-directed motors, i.e., those involved in transport to the cell periphery or to the axon terminus, are known as kinesins [20]. Conventional kinesin is a heterotetramer molecule composed of two heavy chains and two light chains. These motors are highly processive and relatively powerful. Accordingly, only a few motors are required for efficient transport of cargo as large as virus particles. Minus-end transport, i.e., traffic directed from the periphery to the cell center or from the axon terminus to the cell body, is mediated by cytoplasmic dynein [21]. This complex motor protein consists of two dynein heavy chains with ATPase activity, two intermediate chains, and four intermediate light chains. A large protein complex known as dynactin, which also interacts with certain cargo proteins, also enhances the processivity of dynein.

## Conclusions

Since all cells, including neurons, express a relatively diverse range of molecular motors, a major challenge remains to identify which motors transport viral cargo and how viral gene products engage these motor proteins for selective transport. Does one solution for traffic control fit all viruses? Probably not. If we have learned anything over the years, it is that viral genomes have more solutions to cell biology problems than we can imagine at the moment.

The direction taken by virion components in neurons after infection, as well the extent of spread among chains of connected neurons (a neuronal circuit), can be the difference between a minor peripheral infection and lethal brain infection. To spread from presynaptic neurons to postsynaptic neurons, viral components must be sorted to axons (Figure 1). To spread in the other direction (retrograde spread), viral components must be sorted to sites of post-synaptic contact in cell bodies and dendrites. Understanding this type of traffic and its control will provide basic knowledge toward understanding how to block neuronal invasion, spread, and its resulting damage. In addition, as viral tracing of neural circuitry has become an essential tool in the neuroscience community, new discoveries in viral trafficking will be immediately applicable for many ongoing fundamental research projects in neuroscience. I fully expect that these studies will reveal detailed functional insights into neural circuit organization that have not been possible to achieve in the past. Similarly, understanding the basic principles of viral traffic in neurons will advance the utilization of viral vectors for gene delivery and therapy and may ultimately lead to new therapeutic targets.
